# Nanopolymers for magnetic applications: how to choose the architecture?

**DOI:** 10.1039/d2nr01502a

**Published:** 2022-06-16

**Authors:** Deniz Mostarac, Yan Xiong, Oleg Gang, Sofia Kantorovich

**Affiliations:** Faculty of Physics, University of Vienna Boltzmanngasse 5 1090 Vienna Austria deniz.mostarac@univie.ac.at; Research Platform MMM Mathematics-Magnetism-Materials Vienna Austria; Columbia University New York USA; Brookhaven National Laboratories New York USA

## Abstract

Directional assembly of nanoscale objects results in morphologies that can broadly be classified as supra-molecular nanopolymers. Such morphologies, given a functional choice of the monomers used as building blocks, can be of ubiquitous utility in optical, magnetic, rheological, and medical applications. These applications, however, require a profound understanding of the interplay between monomer shape and bonding on one side, and polymeric properties – on the other. Recently, we fabricated nanopolymers using cuboid DNA nanochambers, as they not only allow fine-tuning of the resulting morphologies but can also carry magnetic nanoparticles. However, it is not known if the cuboid shape and inter-cuboid connectivity restrict the equilibrium confirmations of the resulting nanopolymers, making them less responsive to external stimuli. In this work, using Molecular Dynamics simulations, we perform an extensive comparison between various nanopolymer architectures to explore their polymeric properties, and their response to an applied magnetic field if magnetic nanoparticles are embedded. We explain the impact of monomer shape and bonding on the mechanical and magnetic properties and show that DNA nanochambers can build highly responsive and magnetically controllable nanopolymers.

## Abstract 2

Using simulations, we compare nanopolymers with different architectures and/or magnetic nature of monomers, to reveal that nanopolymers based on DNA nanochambers are a compelling, finely tuneable platform for creating magneto-responsive materials.

## Graphical abstract

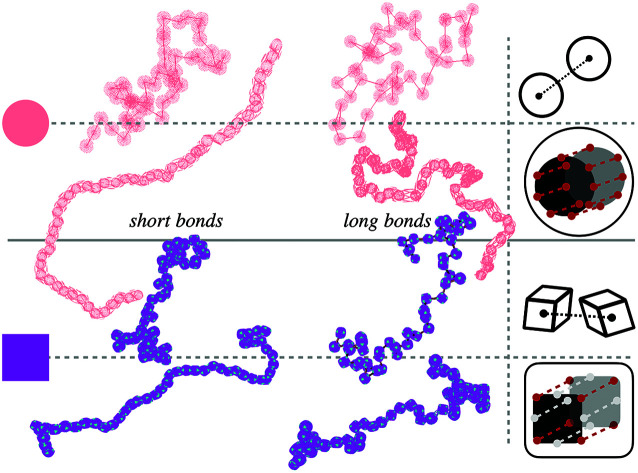

## 1. Introduction

Stimuli-responsive materials are one of the most alluring systems in modern science and one of the central research topics in soft mater physics.^1,2^ Responsiveness to magnetic fields is of particular interest among the plethora of stimuli one can use to modify material properties,^3–6^ in case it is advantageous to have dynamic intensity control and/or great spatial resolution. Furthermore, magnetic fields typically do not interfere with biological tissues and processes, which makes them useful for *in vivo* stimulation of engineered materials.^7^ Polymer-like structures with magnetic nanoparticles (MNPs), commonly referred to as magnetic filaments (MFs) are one of the soft matter systems that emerged in attempts to capitalize on this potential. The elegant simplicity of merging polymer-like systems and MNPs as a solution to the problem of magneto-responsive material design has naturally sparked a great deal of theoretical research. Nanoscopic, polymer-like structures have been shown to exhibit extraordinary plasmonic,^8–13^ magnetic,^14–17^ electronic^18–20^ and mechanical^21,22^ properties. Theoretically, the properties of MFs exposed to external magnetic fields have been mostly explored in bulk.^23–30^ MFs with super-paramagnetic MNPs have been theoretically investigated in artificial swimmer designs.^31–33^ In-field behaviour (*i.e.* buckling, coiling and bending) of MFs with super-paramagnetic MNPs has been investigated under multiple conditions,^34,35^ such as having the MFs grafted to a surface,^36^ or exposed to a rotating or fast precessing magnetic field.^37–39^ MFs in general are promising as a basis for bio-medical application designs.^40–42^ MFs with paramagnetic monomers have been investigated and characterized as potential micro-mixers,^43^ as well as for cargo capture and transport purposes.^44^

**Fig. 1:**
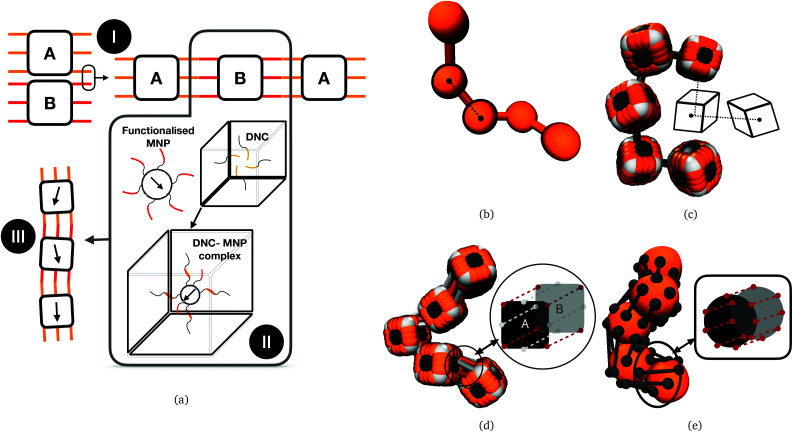
(a) Conceptual illustration relating assembly of DNC nano-objects (top left) to a prospective magnetic filament design (bottom left). I: Bonds between DNC A and B (with orange and red bonds, respectively) hybridize to formDNC nanopolymers. II: DNC can be encoded with anchoring strands to encapsulate functionalised MNPs, forming DNC–MNP complexes; III: prospective DNC–MNP MFs. (b) CTC crosslinking for spherical monomers; (c) CTC crosslinking for cubic monomers; (d) FTF crosslinking of cubic monomers; (e) FTF crosslinking of spherical monomers. (b)–(e) are for visualisation purposes only.

It is important to underline that while there is a breadth of designs of magnetoresponsive micro- and nanoscopic systems that can be nominally considered as magnetic filaments,^45–74^ many of them are fundamentally incompatible with the idea of a polymer-like entity controllable with magnetic fields. A MF as a representative member of highly magneto-responsive, smart nanomaterials, is a compelling system only as far as it has a flexible backbone and a highly tuneable nanostructure. Despite exciting strategies such as exploiting entropy to tailor spatially organized structures of nanoparticles in polymeric systems,^75^ and the amount or research summarized in the paragraph above might suggest, it remains a matter of fact that flexible, nanoscale MFs, with a finely tuneable nanostructure, have not been synthesized yet. The key difficulty in such an endeavour is instilling selective, anisotropic interactions between nano-objects that are otherwise entirely isotropic, with colloids that are chemically stable and when crosslinked, remain so permanently. Thus, even though a lot of variously shaped magnetic nanoparticles are currently available,^76^ how to efficiently crosslink them remains a challenge. To this end, DNA nano-objects have emerged as one of the most prominent candidates, due to their structural programmability and selectivity of sequence-specific interactions.^77–93^ Assembly of such DNA nano-objects offers an attractive route for forming 1D array, polymer-like morphologies – nanopolymers, composed of nano-sized monomers.^91,92,94–105^ Recently, we explored the phase diagram of divalent cuboid DNA nanochambers (DNCs) as a function of bond design, length and number of DNA linkages involved in the inter-cuboid connectivity. DNC nano-objects are created by DNA origami technology. They consist of four double-layer DNA helix walls (outer layer: 12 × 12 double helices, inner layer: 10 × 10 double helices) that enclose a cavity (25 nm × 25 nm × 28 nm). Furthermore, ssDNA linkers with sequence-based specificity placed at a pair of opposite faces of the cubic DNC surface (meaning front and back face) form bonding sites, facilitating directional (lateral) inter-object interactions. We have shown that DNCs can form nanopolymers and can be used as templates for targeted assembly of nanoparticles.^92,106^ A schematic depiction of DNCs with complimentary bonding sites on their surface, forming nanopolymers, as well using DNCs to incorporate MNPs to form DNC–MNP complexes, resulting in nanoscopic MFs, are shown in Fig. 1(a). Resulting filaments are not conventional polymer-like structures, distinct in both crosslinking and monomer shape. MNPs are encapsulated in cubic DNCs that are connected to each other *via* multiple bonds holding their adjacent faces together. There is a complex interplay between bonding and monomer shape that affects the mechanical and magnetic properties of MFs. Moreover, on the length-scales characteristic to DNCs, depending on the magnetic material, the MNPs used to form DNC–MNP complexes could be ferro- or super-paramagnetic. The type of magnetic relaxation will have a profound impact on magnetic and mechanical properties of the MFs, particularly in conjunction with the interplay between monomer shape and inter-monomer connectivity. Recently published strategy of crosslinking nanostructures under the influence of an applied magnetic field presented in Kapuscinski *et al.*^107^ and references therein, suggest that DNC–MNP complexes with ferromagnetic MNPs could form MFs where the remnant magnetisation is coaligned with the polymer backbone. Below, step-by-step, we reveal the impact of monomer shape, bonding, and magnetic characteristics of MNPs on the equilibrium properties of nanopolymers, using Molecular Dynamics (MD) simulations. Even though the solvent is not modelled explicitly here, *θ*-solvent conditions are assumed. Comparing nanopolymers with different architectures and/or magnetic nature of monomers, we reveal that DNC-based nanopolymers represent a compelling, finely tuneable platform for creating magneto-responsive materials. In effect, this work establishes guidelines how to efficiently use DNCs to synthesize MFs with desired properties.

## 2. Results

Computational models we use in this study are depicted in Fig. 1(b)–(e). These models are designed to encompass a range of polymeric systems with different monomer shape and crosslinking. Fig. 1(b) and (c) show representations of polymer-like systems, with spherical and cubic monomer shape, respectively, crosslinked centre-to-centre (CTC), meaning that only the translational degrees of freedom of monomers are restricted. Combination of CTC crosslinking and spherical monomer shape corresponds to a self-avoiding walk.^108^ The DNC nanopolymer model shown in Fig. 1(d) captures the distinctive cuboid monomer shape together with the specific inter-monomer connections, we refer to as face-to-face crosslinking (FTF), where for the adjacent faces, A and B, of two cubes, all the corners and side midpoints are bonded. Model depicted in Fig. 1(e), is designed to reproduce the characteristics of FTF crosslinking, in conjunction with spherical monomers. Important to note is that FTF crosslinking leads to a stiffer polymer backbone as it couples not only the translational but also rotational degrees of freedom of monomers. In other words, monomers can still rotate, but cannot do so freely with respect to their neighbours along the backbone.

By varying the equilibrium bond length *r*_0_, we vary the equilibrium inter-monomer distance. This corresponds to varying the length design parameter of the self-assembly phase, as discussed in Xiong *et al.*^106^ Details of the implementation of all the above-mentioned realizations are provided in Simulation methods part in Section 4.4.

### 2.1. Polymeric properties of DNC nanopolymers

**Fig. 2:**
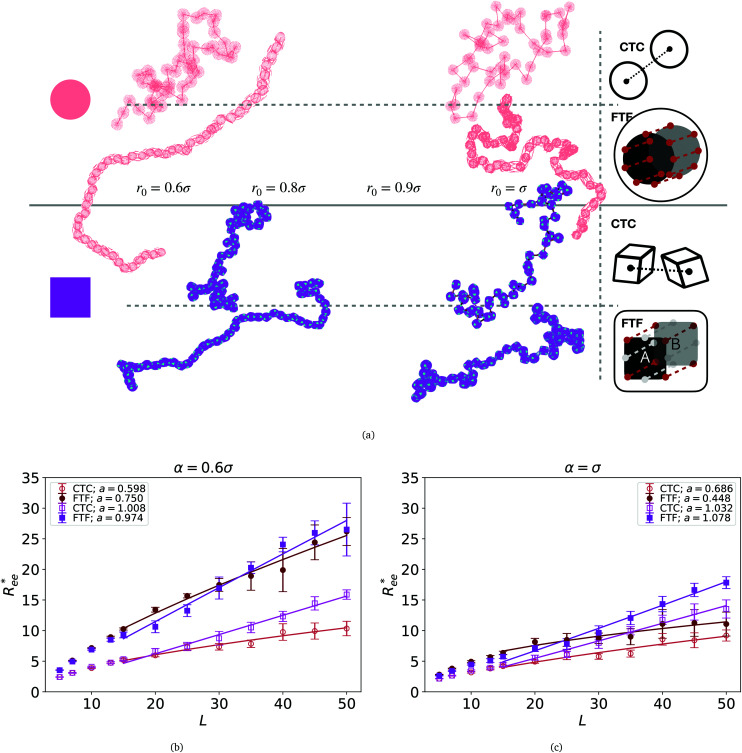
(a) Representative simulation snapshots of nanopolymer conformations, across the combinations of monomer shape, crosslinking approach and *r*_0_ we explored. (b) and (c) show normalized end-to-end distance, 
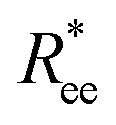
 as a function of monomer number *L*, for different *r*_0_ = *ασ*, where *α* ∈ {0.6, 1}, and *σ* is particle size. Datapoints for nanopolymers with spherical monomers are represented with spherical symbols (red); datapoints for nanopolymers with cubic monomers are represented with square symbols (purple). Filled symbols, shown in a darker color shade, represent nanopolymer models with FTF crosslinking; non-filed symbols, shown in a lighter color shade, are for CTC crosslinking. Error bars are calculated as the standard deviation of 
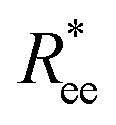
 across 40 independent simulations. Solid lines represent the power-law fits, explained in the text. Values of the fit exponents are shown in the legend. All fit parameters are shown in Table 1.

We start the discussion by considering the polymeric properties of designs shown in Fig. 1, where monomers are not magnetic. We calculate the normalized end-to-end distance, 
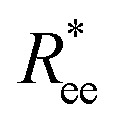
, as the distance between the centres of mass of the first and the *L*-th monomer: 
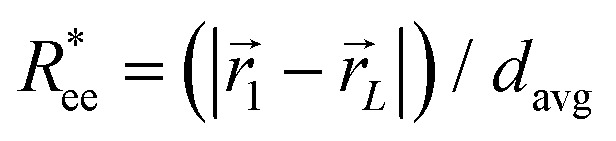
, with *d*_avg_ denoting the average inter-monomer distance between neighbouring monomers along the backbone. The value of *d*_avg_ is calculated separately for each crosslinking approach, monomer shape, *L* and *r*_0_, as the mean value of all nearest-neighbour distances inside a nanopolymer. Our aim here is to underline DNC features, that is, cuboid monomer shape and specific inter-monomer connectivity, and how they lead to unique properties. The results of how monomer shape, crosslinking and *r*_0_, manifest themselves in 
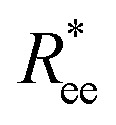
 are shown in Fig. 2.

To help visualize key points in the results presented below, in Fig. 2(a), we show representative simulation snapshots of the conformations our models take, across the combinations of monomer shape, crosslinking approach and *r*_0_ we explored. Independently from value of *r*_0_, presented as increasing from Fig. 2(b)–(c), the most rigid nanoscopic polymer model is the one with FTF crosslinking with cubic monomers, whereas conformations with most coiling are assumed by the nanopolymers with spherical monomers and CTC crosslinking, as is to be expected from a self-avoiding walk. Differences between models are exacerbated for smaller *r*_0_. It is interesting to underline that the shape of the monomers manifests itself only for relatively large values of *L.* Thus, below *L* = 15 the behaviour of the end-to-end distance is defined exclusively by the type of crosslinking. The way 
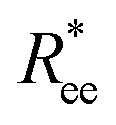
 grows with *L* depends predominantly on the monomer shape. The 
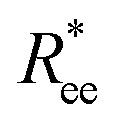
 gradient decreases, with increasing *L*, especially for nanopolymers with spherical monomers. To quantify the differences between the growth rates, for each of the models, we fit the simulation data with *bL*^*a*^ power-law, starting from *L* ≥ 15. These fits are shown in Fig. 2 with solid lines. The fit parameters are collected in Table 1. Nanopolymers with spherical monomers and CTC crosslinking exhibit the scaling of a self-avoiding walk (*a* ∼ 0.5). The situation changes drastically for FTF crosslinking of spherical monomers: the exponent *a* decreases with *α* and never corresponds to a self-avoiding walk. For cubic monomers, 
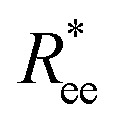
 grows almost linearly with *L* independently from the type of crosslinking. The latter affects the prefactor *b*. Nanopolymers with FTF crosslinking are in general straighter than their CTC counterparts. The closest to unity exponent *a* can be found for a nanopolymer with cubic monomers and CTC crosslinking, with *r*_0_ corresponding to monomer close contact. For nanopolymers with CTC crosslinking, increase in *α* leads to an expected increase of the scaling exponent *a*, regardless of monomer shape. The growth of the equilibrium inter-particle distance can be regarded as an increase of an effective monomer diameter or swelling of the nanopolymer. However, the shape of monomers manifests itself in a nontrivial way when their rotational degrees of freedom are coupled to the backbone. Nanopolymers with FTF crosslinking have difficulties to coil for short bonds (*α* = 0.6). An increase in *α* leads to coiling, where for filaments with spherical monomers, conformations become more compact, and we see a decrease in *a*. However, there is an additional, purely steric coupling between the relative orientation of cubic monomers. In fact, relative orientation coupling between cubic monomers is mostly steric, which is why *a* is so similar for nanopolymers with cubic monomers, regardless of crosslinking. These points are also depicted by the typical conformations presented in Fig. 2(a).

**Table 1:** Parameters obtained by fitting with a power law *y*(*L*) = *bL*^*a*^ for different value of *r*_0_

Bond length	Shape	Fit, CTC	Fit, FTF
*r*_0_ = 0.6*σ*	○	*a* = 0.598, *b* = 1.008	*a* = 0.750, *b* = 1.361
	□	*a* = 1.008, *b* = 0.303	*a* = 0.974, *b* = 0.620
*r*_0_ = 0.8*σ*	○	*a* = 0.665, *b* = 0.740	*a* = 0.523, *b* = 2.228
	□	*a* = 1.071, *b* = 0.230	*a* = 1.018, *b* = 0.412
*r*_0_ = 0.9*σ*	○	*a* = 0.676, *b* = 0.676	*a* = 0.485, *b* = 2.051
	□	*a* = 1.052, *b* = 0.237	*a* = 1.076, *b* = 0.295
*r*_0_ = *σ*	○	*a* = 0.686, *b* = 0.622	*a* = 0.448, *b* = 1.981
	□	*a* = 1.032, *b* = 0.248	*a* = 1.078, *b* = 0.265

One can conclude that shape effects, albeit less pronounced compared to crosslinking effects, are still present. We attribute this to the fact that at *r*_0_ = 0.6*σ*, where *σ* is monomer size, crosslinking ensures that monomers are essentially touching. Most intuitive way to imagine monomer shape effecting 
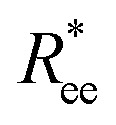
, is the way it restricts monomers sliding past one another or rolling over each other's surface. Spherical monomers can easily do both of those things. Cubic monomers instead are limited in either of the motions when crosslinked. If, however, crosslinking is restrictive enough to minimize such behaviour regardless the monomer shape, 
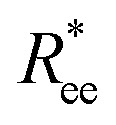
 profiles are grouping, like we see in Fig. 2(b). This explanation is corroborated by persistence length *L*_p_ values, shown in Table 2. We extract *L*_p_ from the decay of the autocorrelation function1
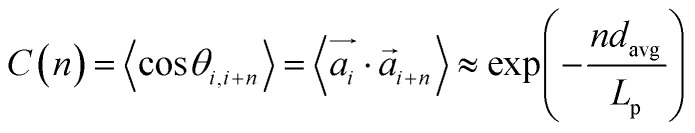
between vectors 
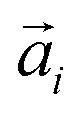
 connecting each pair *i* of neighbouring monomers along the backbone, separated by *n* monomers. Here, we see that models with FTF crosslinking can have several times higher *L*_p_ than their counterparts with CTC crosslinking, where we achieve the highest difference between CTC crosslinked nanopolymers with spherical monomers, and FTF crosslinked nanopolymers with cubic monomers (DNC nanopolymer). Previously, it has been shown that the intricate relationship between the magnetic nature of monomers and crosslinking can result in notably different structural properties and responsiveness of MFs to external magnetic fields, underlining that the crosslinking mechanism strongly affects both magnetic and structural properties.^15^ Considering the peculiarities of DNC nanopolymer crosslinking and the fact that monomer shape affects magnetic properties even in non-crosslinked systems,^109,110^ we expect an interesting shape-crosslinking interplay once MNPs are incorporated in DNCs.

**Table 2:** Persistence length *L*_p_ for different equilibrium length of bonds and monomer shape for nanopolymers, shown in units of *σ* fits performed on datasets for nanopolymer models with *L* = 50

Bond length	Shape	*L*_p_, CTC	*L*_p_, FTF
*r*_0_ = 0.6*σ*	○	1.08	8.41
	□	1.37	8.73
*r*_0_ = *σ*	○	1.06	2.09
	□	1.31	2.24

### 2.2.2. Polymeric and magnetic properties of DNC nanopolymer based MFs

In this section, we present how the magnetic nature and shape of FTF crosslinked monomers, relate to the response to external magnetic fields of MFs with 20 monomers. We compare these results to the magnetic response of a reference filament design with ferromagnetic MNPs, crosslinked with a single bond (see eqn (14)) between each monomer pair, that couples both the translational and rotational degrees of freedom of monomers. We refer to this as constrained crosslinking, in line with ref. 15.

Note that, keeping the analogy to the prospective experimental system, independently from the monomer shape, which is realized in terms of steric interactions (see Section 4.2), the magnetic core is assumed to be spherical in all models. However, considering the actual size of the DNCs, we allow for the possibility of a spherical MNP to be ferro- or super-paramagnetic, abbreviated below to SPM and FM correspondingly. This way, we allow for the possibilities that without a magnetic field applied, MFs can be non-magnetic or have a remnant magnetization.

**Fig. 3:**
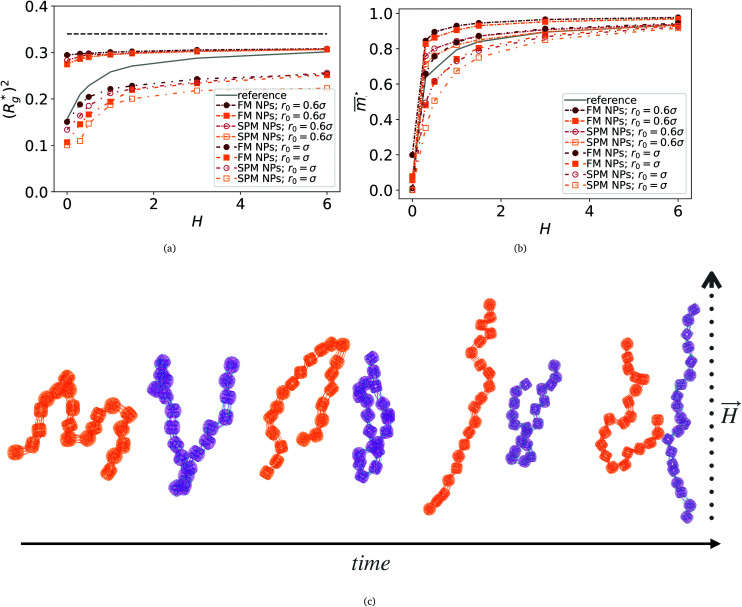
Comparison between models with FTF crosslinking: (a) 
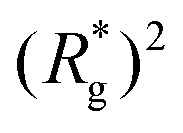
*versus H*. Black dashed line corresponds to the 
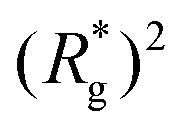
 of a fully elongated filament conformation. (b) 
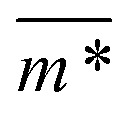
*versus H*. Data for MFs with spherical monomers are represented with spherical symbols (can); data for MFs with cubic monomers are represented with square symbols (orange). Filled symbols, shown in a darker color shade, represent MFs with ferromagnetic monomers; non-filed symbols, shown in a lighter color shade, represent MFs with super-paramagnetic monomers. Reference system of filament with ferromagnetic monomers and constrained crosslinking^15^ is shown with a grey, full line. Errorbars are comparable to symbol size and are as such not shown. (c) Showing typical conformations occurring during the runtime of two separate simulations (orange and purple are meant to distinguish conformations found in independent simulation runs performed for the same system), revealing bend backbone conformations of DNC MFs with super-paramagnetic monomers and *r*_0_ = *σ*.

In Fig. 3(a), we plot the square of the normalized gyration radius 
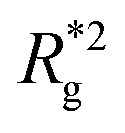
, defined as:2
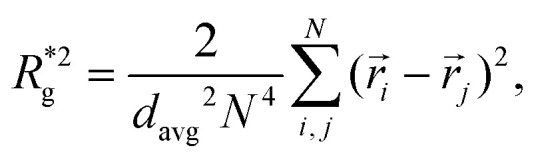
as a function of external magnetic field strength, 
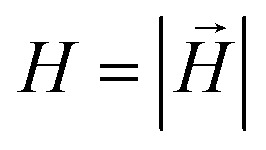
. Here, 
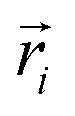
 is the position vector of the *i*-th filament monomer in the lab coordinate frame and *d*_avg_ is an average inter-monomer distance as discussed above. Comparing qualitatively across Fig. 3(a), the difference between overall 
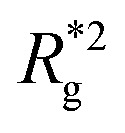
 profiles for different *r*_0_ is stark. For *r*_0_ = 0.6*σ* the profiles are basically flat, regardless of monomers shape and the values of 
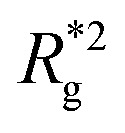
 are higher than for the reference system. Results for *r*_0_ = *σ*, reveal a rapid, field induced increase. Longer bonds make the shape effects visible. MFs with cubic monomers, due to lower inter-monomer correlations inherent to monomer shape anisotropy, have lower 
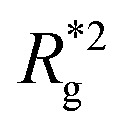
 profiles overall, compared to their counterparts with spherical monomers. Interestingly, having super-paramagnetic monomers further diminishes inter-monomer correlations, compared to their ferromagnetic counterparts.

We continue our investigations of filament response to external magnetic fields, by analysing the average value of the normalized projection of filament magnetic moment, *m̄*, on to the direction of 
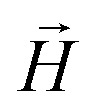
, 
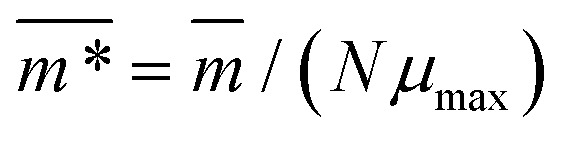
, with *μ*_max_ denoting the maximum dipole moment of a monomer. In accordance with Fig. 3(a) and (b) reveals that larger values of *r*_0_ result in a lower magnetization. Still, as apparent in the magnetization profiles, relatively low to moderate external magnetic fields are sufficient for MFs to be magnetized to saturation. For lower *r*_0_ = 0.6*σ*, FTF crosslinking is restrictive enough to keep the monomers and dipole moments well oriented along 
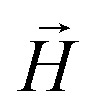
, regardless of monomer shape, or magnetic nature of MNPs. MFs with super-paramagnetic monomers have an overall lower magnetization, because of the effect of local dipole fields between neighbouring monomers, that introduce fluctuations of the direction of the individual dipole moments within the filament. Instead, MFs with ferromagnetic monomers as can be seen from Fig. 3(b) are basically rod-like compared to the rest of the models presented. As *r*_0_ increases, monomers can move more freely and dipole moment orientations can fluctuate more, compared to the MFs with lower *r*_0_, which leads to an overall decrease in magnetization profiles. The shape anisotropy of cubes, on average, further inhibits the ability of dipole moments to predominately align with 
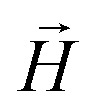
. This in turn means that cubic monomers crosslinked with FTF crosslinking, that is DNC MFs, while generally more elongated, have monomer dipole moments that fluctuate more than their counterparts with spherical monomers. Therefore, an increase in *r*_0_, leads to a more pronounced magnetization decrease in MFs with cubic monomers than for those made of spherical monomers. In fact, magnetization profiles for MFs with spherical, super-paramagnetic monomers and MFs with cubic, ferromagnetic monomers are basically indistinguishable.

In summary, Fig. 3 underlines an effect that permeates the results presented in this work, namely the decorrelation of dipole monomer orientation in MFs with cubic monomers. DNC MFs have monomer dipole moments that locally fluctuate more. Zeeman coupling can compensate for this effect for all models, but DNC MFs with super-paramagnetic NPs that do not fully stretch for high *H*. Instead, as it is shown in Fig. 3(c), we captured several instances of persisting conformations of DNC MFs with a bent backbone. This bending is a signature of MFs with super-paramagnetic spherical monomers^15^ that is clearly intensified by the cubic shape. In the next subsection we analyse whether the relative drawbacks we underlined – less magnetically correlated monomers and more persistent bent backbone conformations – affect the mechanical response of DNC nanopolymer based MFs.

### 3. In-field compression of DNC nanopolymer based MFs in a slit geometry

**Fig. 4:**
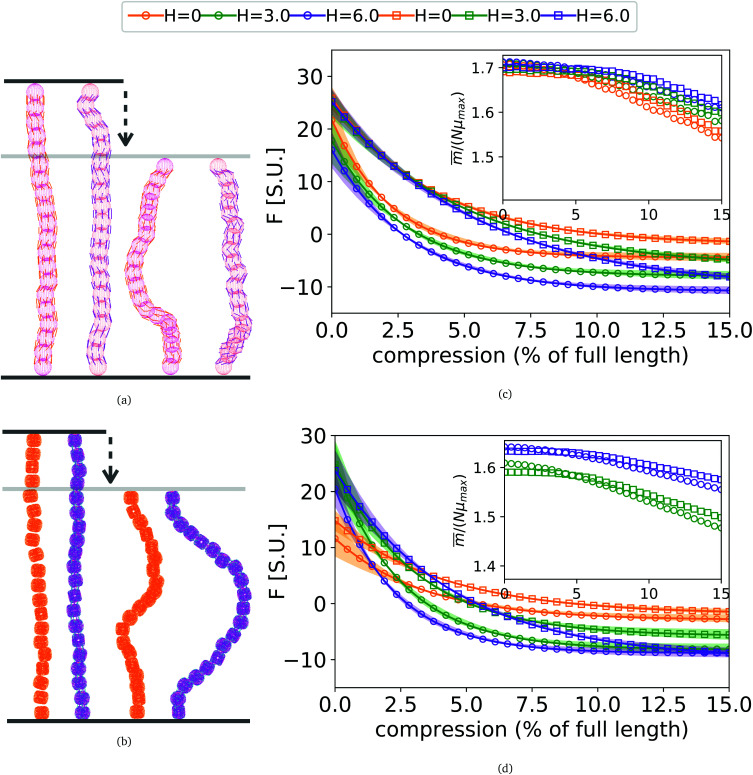
(a) Sketch showing the compression of FTF crosslinked MFs with spherical monomers. (b) Sketch showing the compression of FTF crosslinked MFs with cubic monomers. The upper wall of the slit moves downwards, the field 
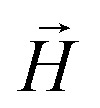
 is pointing up if applied. (c) and (d) showing force projection–compression curves for FTF crosslinked MFs with ferromagnetic and super-paramagnetic monomers, respectively. Exponential fits are plotted. Errors shown as confidence intervals, matching color halos around each force profile. Insets are showing the projection of the magnetization on the compression axis, at equilibrium of confined FTF crosslinked MFs with (c) ferromagnetic; (d) super-paramagnetic monomers, respectively, as a function of compression.

As depicted in Fig. 4(a) and (b), we fix the ends of fully stretched FTF crosslinked MFs with 20 monomers (length of all bonds is their equilibrium length *r*_0_), with different monomer shape and/or magnetic nature of colloids, on the surface of two semi-infinite slit walls. By moving one of the slit walls vertically towards the other, in steps Δ*d*, we elucidate the effects of confinement on MFs and their response to compression, with and without an external magnetic field pointing antiparallelly to the direction of compression. A detailed explanation of the simulation protocol can be found in Section 4.4. The initial, fully stretched conformation (no compression) of a filament is entropically disadvantageous. Indeed, this is confirmed by Fig. 3(a), where one can notice that the 
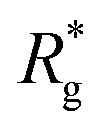
 never approaches that of a rod, 
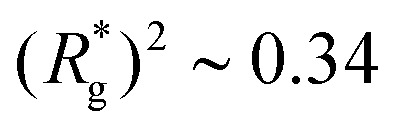
 As a result of this, looking at Fig. 4(c) and (d), the projection on the axis of compression of the force a filament exerts the bottom wall is positive. This shows that the filament is pulling on the bottom wall. With further compression, entropy grows as the filament is given more freedom to fluctuate. At a certain compression, *d*_C_, the filament can reach conformations corresponding to its equilibrium 
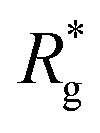
, shown in Fig. 3(a). For such conformations, no net force is exerted on to the walls, and the force-displacement curves cross zero. Looking at orange curves in Fig. 4(c) we compare MFs with ferromagnetic spherical monomers (circles) to their counterparts with cubic ones (squares), without an external magnetic field applied. We see that they stop pulling on the wall at different *d*_C_. MFs with spherical monomers have a slightly higher 
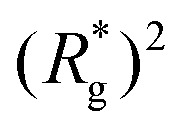
 (see Fig. 3(a)) than their counterparts with cubic monomers. Therefore, they do not need to be given as much leeway to be able to reach equilibrium (R^*^_r_)^2^ corresponding a freely moving filament. For further displacement of the top wall in the range we explored, MFs with ferromagnetic monomers, regardless of monomer shape, oscillate around the zero position.

Switching on the external magnetic field, depicted by green (*H* = 3) and violet (*H* = 6) curves, we see the ramifications of correlations induced by Zeeman coupling. Stretched out configurations with head-to-tail dipole arrangements are facilitated by Zeeman coupling leading to smaller values of *d*_C_. However, given that the 
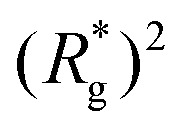
 profiles of freely moving MFs with spherical monomers and *r*_0_ = 0.6*σ* are basically flat, only a small difference between *d*_C_ at any strength of magnetic field applied is observed. For their counterparts with cubic monomers, a difference can be noticed if the field is switched on. This is in line with the small 
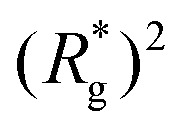
 increase observed in Fig. 3(a). On further compression past *d*_C_, the force becomes repulsive, corresponding to MFs pushing on to the bottom wall. Notice that for MFs with cubic monomers, the repulsive regime is reached for larger compression, than for their counterparts with spherical monomers. This is entirely in line with the dipole de-correlation effect we referred to earlier.

Force-compression curves for MFs with super-paramagnetic monomers, shown in Fig. 4(d), exhibit qualitatively the same trends seen in Fig. 4(c). The most apparent difference, however, is a strong dependence of *d*_C_ on *H* for MFs with the same monomer shape. This can be understood from the magnetization curves in Fig. 3(b), where for MFs with ferromagnetic monomers, *H* = 3 is already sufficiently strong to reach magnetization saturation, while for MFs with super-paramagnetic monomers it is not.

Looking at insets of Fig. 4(c) and (d), showing the projection of the magnetization on the vertical axis, of the non-immobilized part of the filament, at equilibrium for MFs with ferromagnetic and super-paramagnetic monomers respectively, we see a crossover between two linear magnetization regimes. The change of the regimes happens at corresponding values of *d*_C_. The differences between ferro- and super-paramagnetic monomers is manifested in the absolute scale of the magnetization before *d*_C_. Past *d*_C_, the growing separation between the magnetization curves reflects the competition between entropy on one side and dipole–dipole interactions with Zeeman coupling, on the other.

## 3. Conclusion

In this work, using computational models, we compare nanopolymers with different architectures and/or magnetic nature of monomers. It turns out that cubic monomer shape makes nanopolymers more rigid only if the inter-monomer bonds are short enough and the number of monomers is rather high. For moderately long nanopolymers (15 < *L* < 30) with comparable crosslinking, end-to-end distance largely similar regardless of monomer shape.

Apart from varying monomer shape, we also consider different types of crosslinking: we investigate the differences between nanopolymer designs with centre-to-centre and face-to-face crosslinking. The latter is of particular interest as it represents well recently synthesized nanopolymers based on cubic DNA nano-chambers (DNCs). As expected, face-to-face crosslinking results in more linear conformations and for short bond length can double the equilibrium end-to-end distance, if number of monomers in the nanopolymer is large enough.

Next, using the aforementioned model, we demonstrate that DNC nanopolymers represent a compelling, finely tuneable platform for creating magneto-responsive materials. We are encouraged by the fact that, even the lowest magnetization we observed for filaments based on DNCs at a given applied field is still significantly higher than that of conventional magnetic nanoparticle suspensions (ferrofluids). Furthermore, the magnetic fields needed for a DNC-based magnetic filament to reach magnetic saturation are relatively low, as the thermal fluctuations are hindered by both monomer shape and multiple inter-monomer bonds. We show that the steric penalty brought by cubic monomer shape to initial susceptibility can be compensated with bond length: if the bonds are short enough, the initial slope of magnetization curves for nanopolymers made of spheres and cubes basically coincide. In general, cubic monomer shape slightly diminishes magneto-responsiveness, as quantified by magnetization, most pronounced for DNC MFs with super-paramagnetic monomers. While notable, the difference to their counterparts with spherical monomers is not high enough to discourage the use of DNC nanopolymers for filament designs with super-paramagnetic MNPs. Quite on the contrary, it opens the door to interesting phenomenology related to backbone bending noted for MFs with super-paramagnetic monomers.

Finally, we investigate the mechanical resistance to compression that MFs with different architecture and/or magnetic nature of monomers exhibit, by grafting two filament ends on slit walls, one of which is then moved towards the other. Compared with filament designs with spherical monomers, cubic monomer shape of DNC MFs proves to be advantageous, with a smoother and more controllable resistance to compression.

Currently, we are investigating rheology and dynamics of the systems introduced here, as one would expect the shape of the monomers to affect hydrodynamic behaviour of the nanopolymers.

## 4. Simulation methods

In this section we explain in detail the general computational scheme, interactions and computational models used in this work.

### 4.1. General scheme

We performed Molecular Dynamics (MD) simulations in the ESPResSo software package.^111^ The effects of the background fluid were handled implicitly, *via* the Langevin thermostat^112^ at fixed temperature *T*. In practice it means that the Langevin equations of motion are integrated over time *t* numerically:3
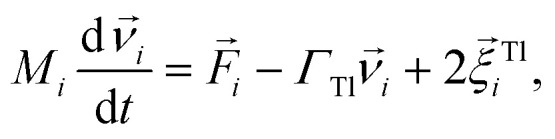
4
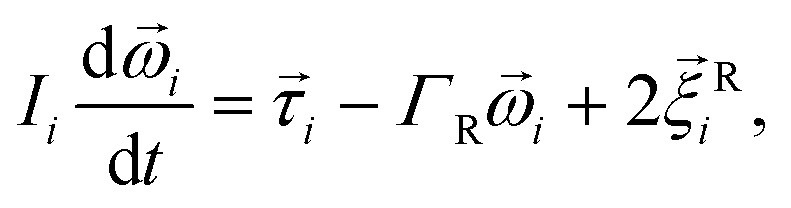
where for the *i*-th particle in eqn (3), *M*_*i*_ is the mass tensor, 
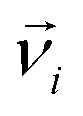
 denotes the translational velocity, 
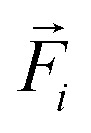
 is the force acting on it, *Γ*_Tl_ denotes the translational friction coefficient, 
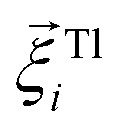
 is a stochastic force modelling the random forces of the implicit solvent. In eqn (4), *I*_*i*_ denotes *i*-th particle inertia tensor, 
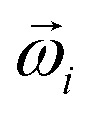
 is its rotational velocity, *τ*_*i*_ is torque acting on it, *Γ*_R_ denotes the rotational friction coefficient, and the 
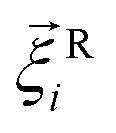
 is a stochastic torque serving for the same purpose as 
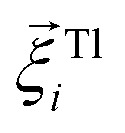
. Both stochastic terms satisfy the conditions on their time averages:^113^5
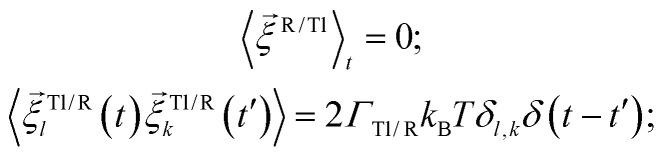
where *k*,*l* = *x*,*y*,*z*.

Forces and torques in eqn (3) and (4) are calculated from inter-particle interaction potentials. In all our simulations here, we used no periodic boundary conditions, as the focus is always on a single polymer-like chain per simulation box. For the integration, velocity Verlet algorithm was used.^114^

### 4.2. Raspberry model and steric interactions

If particles are spherical and we are not interested in their surface properties, but rather in their effective excluded volume, the typical approach to model their steric repulsion in MD is to use Weeks–Chandler–Andersen pair potential (WCA):^115^6
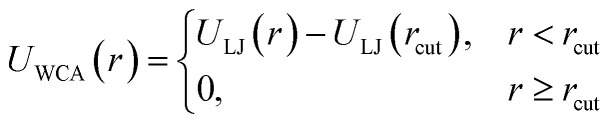
where and *U*_LJ_(*r*) is the conventional Lennard-Jones potential:

7 *U*_LJ_(*r*) = 4{(*σ*/*r*)^12^ − (*σ*/*r*)^6^}

where *σ* is the characteristic diameter of the sphere and the cut-off value is *r*_cut_ = 2^1/6^*σ*. Parameter *ε* defines the energy scale of the repulsion.

For non-spherical particles, or spherical particles whose surface properties matter, there are two ways of modelling steric interactions: the first one is on each MD integration step to solve an algebraic system of equations to check for the overlap of complex shapes; the second one is to build the particle complex shape out of spherical beads – WCA centres of required size *σ*. The second method is less accurate but is much faster. The particle resulting from the second method is usually addressed as a raspberry particle.^116^ This is how we construct the cubes. Positions, radii, and parameters for the steric interactions between the WCA-spheres that make out cubic-shaped monomers are calculated using the superball model for *q* = 2, developed by Donaldson *et al.*,^110^ where *q* is the shape parameter in the superball equation given by:8
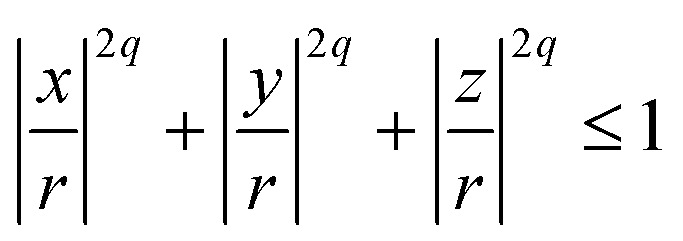


If *q* → ∞, eqn (8) is describing a cube with perfectly sharp edges with its centre in (0,0,0). With *q* = 1, eqn (8) describes a sphere with radius *r* centred at the origin.

ESPResSo makes use of the concept of virtual sites.^111^ The virtual particle or site is fixed with respect to the reference frame of the real particle to which it is attributed. It is possible to define any number of virtual sites at any position with respect to the reference frame of a given real particle. The interactions between virtual–virtual and virtual–real particles can be specified using any suitable potential. All forces exerted on the virtual particles as a consequence of such interactions are instantaneously propagated to the reference real particle in each time step. These features allow to define rigid bodies with any shape by defining proper arrangements of virtual sites. In Fig. 1(c) and (d) one can see a raspberry representation of cubes employed in this study, that we used successfully for investigating magnetic and charged cubes in previous works.^110,117^

### 4.3. Magnetic interactions

Monomers in this work can be either ferromagnetic or super-paramagnetic. Dipole moments of ferromagnetic monomers or monomers with ferromagnetic MNPs embedded within them, are modelled as central, point-particle dipole moments, 
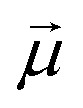
, of a fixed length 
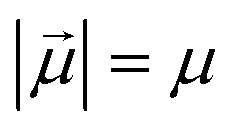
. Long-range magnetic inter-particle interactions are accounted for *via* the standard dipole–dipole pair potential:9
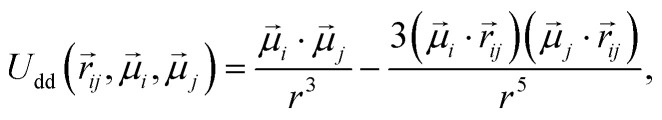
where the inter-particle distance is 
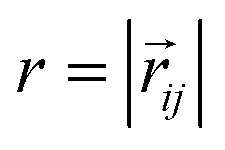
, and 
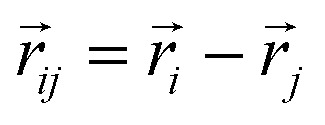
 is the displacement vector connecting *i* and *j* monomer centres with dipole moments 
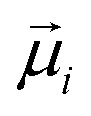
 and 
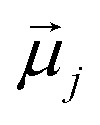
, respectively. Zeeman interactions coming from the presence of an external magnetic field 
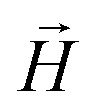
, are realized *via* Zeeman coupling potential:10
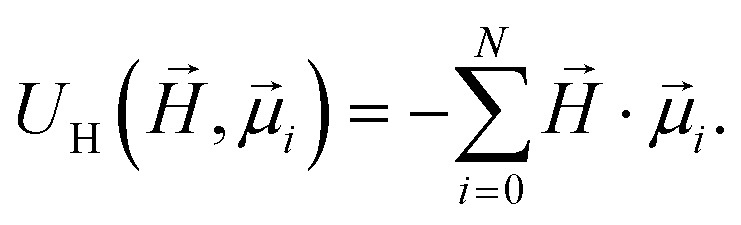


To model the phenomenology of super-paramagnetic MNPs accurately, one needs to calculate the total field 
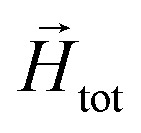
 in each point of the system. The total magnetic field is the sum of 
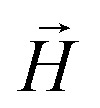
 and the dipole field 
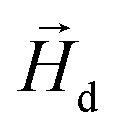
. The latter field, created by particle *j*, at position 
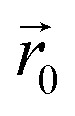
 is given by:11
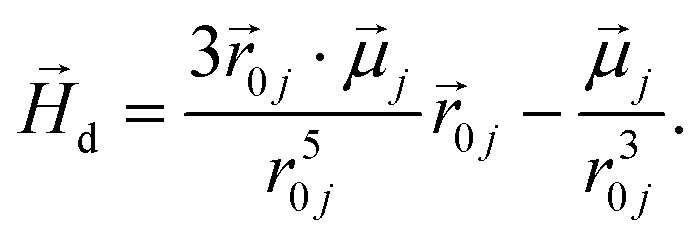


To study of the response of MFs to fields of arbitrary strength, we define the dipole moment 
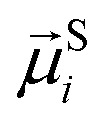
, of an *i*-th super-paramagnetic particle at a given temperature *T*, as:12
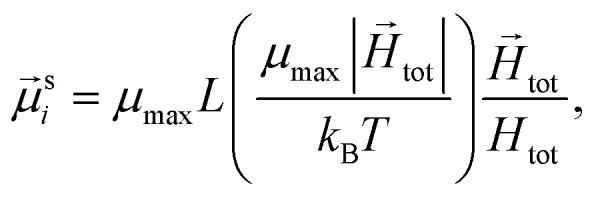
where 
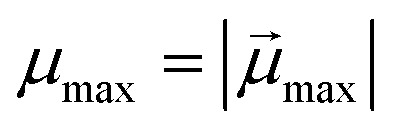
 is the modulus of the maximal magnetic moment, 
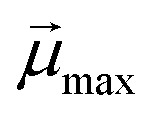
. Here, *k*_B_ is the Boltzmann constant and *L*(*α*) is the Langevin function:13
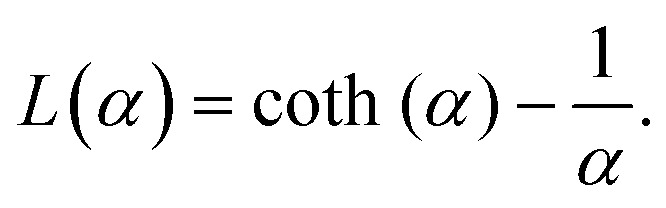


Not only does this approach lend itself to account for nonlinear effects but, the expression (12) is a generalisation of mean-field approaches, such as the modified mean field approach.^118^ The difference here is that we do not need to make any assumption to calculate 
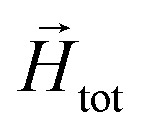
. This approach is also verified by the analytical calculations for super-paramagnetic particle magnetization.^119^

### 4.4. Simulation protocol

As schematically depicted in Fig. 1, we want to highlight the impact of crosslinking together with monomer shape, on the equilibrium properties of resulting polymer-like chains. We model the bonds in our models as finitely extendable springs, described by FENE potential:^120^14
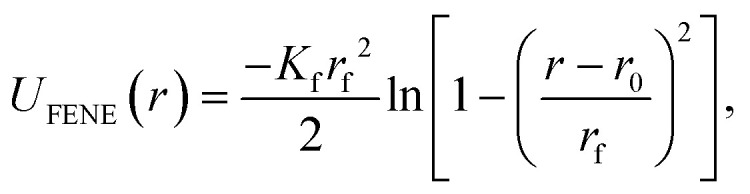
where *K*_f_ is the rigidity of the bond, *r*_f_ is the maximal stretching length and *r*_0_ is the equilibrium bond length of a FENE bond. We discern two fundamentally different ways of joining monomers into polymer-like chains, we refer to as either centre-to-centre (CTC) or face-to-face (FTF) crosslinking.

By CTC crosslinking, it is to be understood that the centres of mass of neighbouring monomers are bonded centre-to-centre *via* FENE bonds as shown in Fig. 1(b) and (c). In this way, we ensure a close contact between the monomers, without restricting their rotations. By FTF crosslinking, we aim to establish a notion of relative orientation of monomers with respect to their nearest neighbours, as well as to couple their rotational degrees of freedom with the backbone.

For chains of spherical monomers, we achieve this by creating two crowns of virtual sites on opposite sides of spherical monomers, that serve as anchoring points for FENE bonds, see, Fig. 1(e). Positions of virtual sites that make the crowns are determined by finding the cross-section of a sphere with diameter *σ* and a plane, whose normal vector is pointing in the direction in along which the backbone is initially spanning. We are looking for circles of points that solve this problem, whose centres are located at *r*_*i*_ + *σ*/4 and *r*_*i*_ − *σ*/4, respectively, where *r*_*i*_ is the position of the centre of the *i*-th monomer. We create 8 equidistant virtual sites on each of the resulting circles on the surface of the spherical monomer. For each pair of monomers, adjacent virtual sites are linked by FENE bonds. In this way we introduce the notion of relative orientation for spherical monomers, and fully couple their rotational degrees of freedom.

For chains of cubic monomers, FTF crosslinking captures the relevant characteristics of the crosslinking that ensues due to the divalent, “polychromatic” nature of *M*_*k*_^l^; *k* = 16 and/or *k* = 64 DNC from Xiong *et al.*^106^ We attach FENE bonds between adjacent corner particles on neighbouring monomers, and between adjacent central edge virtual sites on the faces of neighbouring monomers, schematically depicted in Fig. 1(d).

Timestep by which the equations of motion (3) and (4) propagate the system in all our simulations is 10^−2^. For investigations of end-to-end distance scaling as a function of monomer number, we create chains of *L* monomers, *L*∈[2,50] and place each of them in a separate simulation box initially fully straight and stretched, with the backbone orientated randomly. We run forty parallel, model/length specific simulations, for different values of *r*_0_ at constant *T* = 1. We firstly make sure that system relaxes into an equilibrium configuration, by running an integration cycle for 2.1 × 10^6^ integration steps. After the relaxation cycle, we start recording simulation snapshots every 7000 integrations, to minimise correlations between observed polymer-like chain conformations. The total length of the measurement cycle is 1.05 × 10^7^ integrations.

For models with magnetic monomers, we use the same simulation protocol as above, except for that we ran 15 parallel, model specific simulations, for different values of *r*_0_ and external magnetic field *H*, at constant *T* = 1.

For response of confined DNC MFs to compression, we placed two parallel steric planes and graft on them to opposite ends of a given chain model. Each chain is in a separate simulation box and initially fully straight and stretched. External field direction is perpendicular to steric planes, along the *z* axis. We run ten parallel, model specific simulations, for different values of external magnetic field *H* at constant *T* = 1. Compression was realized by moving the steric planes in increments of 0.1 every 300 000 integrations, until we reached maximum desired compression of five length-scales. With this we ensure that chains reach equilibrium before each compression step.

### 4.5. Reduced units

Interaction potential between a pair of monomers in chains is determined by the interplay between the steric interaction and FENE bonds between them. We match the parameters so, that the magnitude of the interactions between nearest neighbours in different crosslinking scenarios is nearly the same for a given value of *r*_0_. This is achieved by tuning dimensionless simulation parameters. Thus, the diameter of spherical monomers was set to *σ* = 0.91 while, for cubic monomers, diameter of the central particle is set to *σ* = 1. With the choice of *σ* for central particle in the cube, according to the superball model developed by Donaldson *et al.*^110^ for shape parameter *q* = 2, corner virtual sites have *σ* = 0.41 while vertex virtual sites have *σ* = 0.49. Based on interaction potential matching we determined that the energy scale of the steric repulsion between spherical monomers should be 100 times higher than that of cubic monomers. Therefore, for central particles in the cubes and all virtual sites on the shell, constituting cubic monomers, *ε* = 1, while for spherical monomers *ε* = 100. The overall energy scale in our simulations is determined by the choice of the reduced temperature *k*_B_*T* = 1. The reduced characteristic mass of all massive particles in our simulations is taken to be *m* = 1. Given that we are only interested in equilibrium properties, this value does not affect the results, rather the speed of the convergence. However, the tensor of inertia of cube central particle must be set to represent the fact that they carry a shell of virtual sites that reproduce a cuboid shape. Finally, for simulations of polymer-like chains, following results from interaction potential matching we determined that FENE bonds in centre-to-centre crosslinking should be 9 times as rigid as the ones in face-to-face crosslinking. Therefore, for FTF crosslinking, *K*_f_ = 10, while for CTC crosslinking *K*_f_ = 90. Equilibrium length of FENE bonds *r*_0_ = *αr*_min_, was set to be a *α* multiple (*α*∈0.6, 0.8, 0.9, 1.0) of the particle size (*r*_min_ = *σ*). Maximal extension of each FENE bond, *r*_f_, was set to be 3 times the equilibrium bond length *r*_0_.

We consider reduced saturated magnetic moment of *μ*_max_^2^ = 3, for a range of reduced external magnetic fields *H* ≤ 6. Given a choice of a particular magnetic nanoparticle, such as using magnetite nanoparticles coated with a thin layer of stabilising agent (*i.e.*, oleic acid coating, 2 nm thick), *σ* corresponds (not uniquely) to a colloid with a magnetic core of 15 nm with a dipole moment of 8.5 × 10^−19^ A m^2^. This also means that the maximum of the applied magnetic field range we explored represents moderate fields of only 0.072 T.

## Conflicts of interest

There are no conflicts to declare.

## Supplementary Material
